# Molecular Mechanism of Mesenchyme Homeobox 1 in Transforming Growth Factor β1–Induced *P311* Gene Transcription in Fibrosis

**DOI:** 10.3389/fmolb.2020.00059

**Published:** 2020-04-28

**Authors:** Zhiyuan Wei, Chao Han, Haisheng Li, Weifeng He, Junyi Zhou, Hui Dong, Yuzhang Wu, Yi Tian, Gaoxing Luo

**Affiliations:** ^1^Institute of Burn Research, PLA, State Key Laboratory of Trauma, Burn and Combined Injury, The First Affiliated Hospital of Third Military Medical University (Army Medical University), Chongqing, China; ^2^Institute of Immunology, PLA, Third Military Medical University (Army Medical University), Chongqing, China

**Keywords:** Meox1, P311, promoter, transcription factor, fibrosis

## Abstract

Organ fibrosis is characterized by excessive fibroblast, and extracellular matrix and the molecular basis are not fully elucidated. Recent studies have proven that P311, an 8-kDa conserved protein, could promote various organ fibrosis, such as skin, kidney, liver, and lung, partially through upregulating transforming growth factor β1 (TGF-β1) translation. However, the upstream regulators and mechanism of P311 gene regulation remain unclear, although we previously found that cytokines, hypoxia, and TGF-β1 could upregulate P311 transcription. Here, we aimed to elucidate the molecular mechanism of TGF-β1–induced P311 transcriptional regulation, focusing on mesenchyme homeobox 1 (Meox1). In this article, we identified the core promoter of P311 through bioinformatics analysis and luciferase reporter assays. Moreover, we demonstrated that Meox1, induced by TGF-β1, could bind to the promoter of P311 and promote its transcriptional activity. Furthermore, the effect of Meox1 on P311 transcriptional expression contributed to altered migration and proliferation in human dermal fibroblast cells. In conclusion, we identified Meox1 as a novel transcription factor of P311 gene, providing a new clue of the pathogenesis in fibrosis.

## Introduction

P311, also known as neuronal regeneration-related protein (NREP), is an 8-kDa protein with a PEST domain in its N-terminus and is conserved among species (Studler et al., 1993; Miura and Naganuma, 2000). P311 was first reported in the embryonic brain by Studler et al. (1993) and later found to be broadly expressed in various tissues (Fujitani et al., 2004; McDonough et al., 2005; Tan et al., 2010; Reddy et al., 2011; Guimaraes et al., 2015; Yao et al., 2017; Katta et al., 2018; Qi et al., 2018). Accumulating evidence indicates that P311 is involved in a variety of processes, especially in the pathogenesis of fibrosis (Fujitani et al., 2004; Zhao et al., 2006; Reddy et al., 2011; Guimaraes et al., 2015; Yao et al., 2017; Qi et al., 2018). In renal fibrosis, P311 acted as a positive regulator through transforming growth factor β1 (TGF-β1)/*Drosophila* mothers against decapentaplegic protein (Smads) signal pathways (Yao et al., 2017; Qi et al., 2018). And in lung fibrosis, P311 was related to alveolar regeneration, and its absence was interrelated to human emphysema (Zhao et al., 2006). More importantly, our previous studies showed that P311 was significantly increased in hypertrophic scars among burned patients and that TGF-β1 dramatically induced P311 mRNA expression in human primary fibroblasts (Wu et al., 2004; Zhang et al., 2016).

Two major mechanisms are involved in gene expression regulation, that is, transcriptional and posttranscriptional regulation (Maston et al., 2006; Chen et al., 2017). Promoters are key *cis*-regulatory elements critical for the initiation of gene transcription (Wittkopp and Kalay, 2011). Until now, there have been no reports on the P311 promoter. Moreover, the TGF-β1 pathway can induce many transcription factors (TFs), which play a critical role by binding to the target gene promoters (Kaur et al., 2018). Therefore, we aimed to identify the core promoter of P311 and its specific transacting regulators induced by TGF-β1.

Epigenetic modifications, including DNA methylation and specific histone modifications, are helpful for identifying potential promoters (Heintzman et al., 2007). Specifically, promoters exhibit high levels of histone H3 lysine 4 trimethylation (H3K4me3), H3K4 dimethylation (H3K4me2), and DNase enrichment and a low level of H3K4 monomethylation (H3K4me1) (Heintzman et al., 2007). These modifications provide us with evidence to locate specific promoters, which need to be further validated.

In this study, we identified a novel P311 promoter located at chr5:111999065-111999465 of the hg38 assembly of the human genome through epigenetic modification prediction with the Roadmap Epigenomics Project database and carried out biological validation with reporter assays. Further evidence suggested that mesenchyme homeobox 1 (Meox1), but not Smad2/3/4 (Smads) induced by TGF-β1, significantly increased the mRNA expression of P311 by directly binding to its promoter in human dermal fibroblast–adult (HDF-a) cells. Finally, we found that Meox1 was important for HDF-a proliferation and migration.

## Materials and Methods

### Cell Culture and Treatment

The HDF-a human fibroblast cell line was obtained from American Type Culture Collection and grown in RMPI medium (Gibco, Carlsbad, California, United States). The 293T embryonic kidney cell line was obtained from the Chinese Academy of Sciences and grown in Dulbecco modified eagle medium (HyClone, Logan, Utah, United States). All the media were supplemented with 10% fetal bovine serum (FBS; Gibco) and 1% antibiotics, and cells were grown in a humidified atmosphere with 5% CO_2_ at a temperature of 37°C.

For the TGFβ1 stimulation experiments, 10 ng/mL exogenous TGF-β1 (PHG9214; Life Technologies, Carlsbad, California, United States) was added to the media at 60% cell confluence, which were then continuously cultured for 72 h at 37°C in 5% CO_2_.

### Cloning of Test Fragments

Promoter-1 and a series of shortened fragments were amplified by polymerase chain reaction (PCR) from human genomic DNA using Prime STAR Max DNA polymerase (TaKaRa, Kusatsu City, Japan). Promoter-1 and its shorted fragments were cloned into pGL3-basic vector with *Kpn*I and *Xho*I. Promoter-2 and its shorted fragments were cloned into pGL3-Basic vector with *Kpn*I and *Sma*I (Promega, Madison, Wisconsin, United States). The pGL3 vector containing 0.4-kb promoter without Meox1 binding site (pro-0.4-ΔMeox1) was constructed by Tsingke Biotechnology Corp, Beijing, China. All sequences cloned into the pGL3 vector were verified by sequencing. The PCR primers are shown in Supplementary Tables S1, S2.

The open reading frame sequences of Meox1/Smad2/3/4 were inserted into pcDNA3.1 eukaryotic overexpression vector, which was constructed and verified by Tsingke Biotechnology Corp.

### Transfection and Dual-Luciferase Assays

The small interfering RNAs (siRNAs) in this article were purchased from RiboBio Corp, Guangzhou, Guangdong, China. The antisense and sense siRNA sequences are shown in Supplementary Table S3. According to the manufacturer’s instructions, siRNAs were transfected into cells with Lipofectamine 2000 (Thermo Fisher Scientific, Inc., Carlsbad, California, United States).

All the vectors were transfected into 293T cells or HDF-a cells using Lipofectamine 2000 in Opti-MEM (Thermo Fisher Scientific, Inc., Carlsbad, California, United States). After 24 h of transfection, the cells were collected or cultured as needed. For luciferase activity detection, cells were tested using a dual-luciferase reporter assay system (Promega) according to the manufacturer’s instructions.

### Online TF Prediction

In this study, JASPAR^1^ was used to predict the potential TFs of sequences in *Homo sapiens*. The relative profile score threshold was set to 85%.

### DNA/RNA Extraction, cDNA Synthesis, and Quantitative Reverse Transcriptase–PCR

Total genomic DNA was extracted from 293T cells with a Tiangen DNA extraction Kit (Tiangen, Beijing, China). Total RNA was extracted with TRIzol (Invitrogen, Carlsbad, California, United States), and TB Green-based real-time PCR was carried out with first-strand cDNA synthesis products generated from total RNA (TaKaRa). Relative mRNA expression was analyzed with the ΔΔCt method (Livak and Schmittgen, 2001). Sequences of the reverse transcriptase–quantitative PCR (RT-qPCR) primers of target genes are shown in Supplementary Table S4.

### RNA Sequencing

Total RNA was extracted from stimulated and unstimulated HDF-a cells by TGF-β1. Then, RNA quality was determined with the 2100 Bioanalyzer (Agilent, Carlsbad, California, United States) and quantified using a ND-2000 (NanoDrop Technologies, Carlsbad, California, United States). RNA-sequencing (RNA-seq) was performed by Shanghai Majorbio Bio-pharm Biotechnology Co., Ltd. (Shanghai, China) (Robinson et al., 2010).

### Chromatin Immunoprecipitation–qPCR

Chromatin immunoprecipitation (ChIP) was performed using a ChIP assay kit (Beyotime, Shanghai, China) and Dynabeads Protein G (Invitrogen) as described previously (Mehra and Wells, 2015). The qPCR primer sequences used are shown in Supplementary Table S5. Antibodies against Smad2 (ab71109; Abcam, Cambridge, England), Smad3 (ab28379; Abcam), Smad4 (AF2097, R&D), Meox1 (ab75895; Abcam), polymerase II (ab5095; Abcam), and immunoglobulin G (IgG) (ab2410; Abcam) were used for ChIP assays.

### Western Blotting

Western blotting was performed as described previously (Li et al., 2016). Glyceraldehyde-3-phosphate dehydrogenase (GAPDH) was used as the loading control. Protein bands were visualized using a chemiluminescent detection system (Thermo Scientific), and images were captured with a scanner. Antibodies against Meox1 (ab75895; Abcam) and GAPDH (ab181602; Abcam) were used for Western blotting.

### Scratch Wound-Healing and Transwell Assays

For the scratch wound-healing assay, cells at logarithmic growth phase were transfected with siRNA or treated with pcDNA3.1-Meox1. Then, 2 × 10^5^ cells were seeded into six-well plates and cultured to 70% confluence. Scratch wounds were created in the monolayer (0 h) with 1 mL pipette tips and then captured every 12 h using a Zeiss video microscope Carl Zeiss AG is in Jena, Germany. The wound-healing distance was measured with ImageJ v1.48 software (National Institutes of Health, Bethesda, Maryland, United States).

Transwell assays were carried out with 24-well Transwell plates (8-μm pore size; Millipore, Bedford, Massachusetts, United States); 1 × 10^5^ cells transfected before were seeded on the upper chamber in serum-free medium, whereas the lower chamber contained medium with 20% FBS applied as a chemoattractant. After incubation for 24 h, the cells on the bottom surface of the filter were fixed with 4% paraformaldehyde, stained with hematoxylin-eosin dye, and counted.

### Cell Proliferation Assays

A total of 2,000 cells per well were seeded in 96-well plates after transfection, and cell proliferation was measured by Cell Counting Kit-8 (CCK-8; Beyotime) assay according to the manufacturer’s instructions. Proliferation rates were determined at 12, 24, 36, and 48 h after seeding by measuring the absorbance at 450 nm with a microplate reader (Bio-Rad, Hercules, California, United States).

### Statistical Analysis

Data are expressed as the mean ± SD. Comparisons of wound distances and cell growth were carried out with one-way analysis of variance via SPSS 18.0 belongs to International Business Machines Corporation (IBM), which located in Al monk, New York, United States. Differences between groups were determined by Student *t* test via GraphPad Prism 5.0 belongs to GraphPad Software Inc. which is in San Diego, California, United States. Differences for which *P* < 0.05 (two-sided) were considered statistically significant (^∗^*P* < 0.05; ^∗∗^*P* < 0.01; and n.s., not significant).

## Results

### Epigenetic Modifications of Two Potential Promoters of the NREP (P311) Gene in Primary Foreskin Fibroblast Cells

**FIGURE 1:**
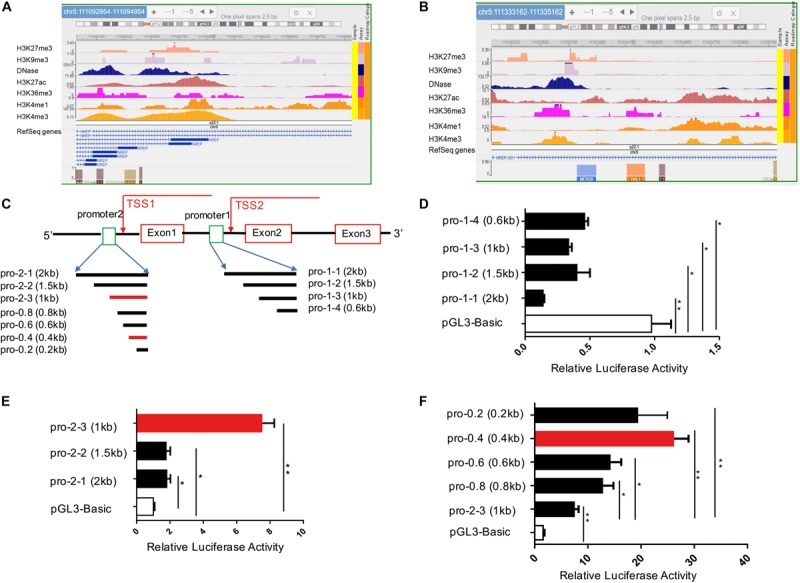
Identification of a novel P311 promoter with epigenetic modifications and the dual-luciferase reporter assay. **(A,B)** Levels of epigenetic modifications of two potential promoters of P311, chr5:111092954-111094954 (**A**, promoter-1) and chr5:111333162-111335162 (**B**, promoter-2) (reference genome: GRCh37/hg19), determined with the Roadmap Epigenomics Project database. **(C)** Sketch map of the P311 gene, with the gene indicated as a thick line. Rectangular boxes indicate exons. Square boxes indicate the P311 promoters including promoter-1 (pro-1-1) and promoter-2 (pro-2-1). Transcriptional start sites (TSSs) are marked with arrows. All the truncated promoters are shown in **(A)** and were cloned into the pGL3-Basic vector. **(D,E)** Luciferase activities in 293T cells transfected with the indicated reporter constructs containing various truncated promoters determined by dual-luciferase reporter assay. Promoter-1 was truncated into four fragments **(D)**. Promoter-2 was truncated into 3 fragments **(E)**. **(F)** The luciferase activities in cells transfected with four truncated fragments of pro-2-3. All truncated fragments were cloned into pGL3 vectors. Error bars show the mean ± SD. *P ≤ 0.05; **P ≤ 0.01; n.s., not significant; Student *t* test. Three independent experiments were carried out.

To identify potential promoters of the human P311 gene within its 5′ region, we reviewed all 14 transcripts of the P311 gene in the University of California–Santa Cruz genome browser^2^. Due to diversity in the transcriptional start site (TSS), we separated all 14 transcripts into two groups with two different potential promoters, promoter-1 (chr5:111092954-111094954) and promoter-2 (chr5:111333162-111335162), of the hg19 assembly of the human genome. Because H3K4me3 and DNase are the most important epigenetic markers of an active gene promoter, we utilized the Roadmap Epigenomics Project database^3^ to analyze promoter-1 and promoter-2 in human foreskin fibroblast samples for the following markers: DNase, H3K4me1, H3K4me3, H3K27ac, H3K9me3, and K3K27me3 (Heintzman et al., 2007). As shown in Figures 1A,B and Supplementary Figure S1, the active promoter-associated epigenetic markers H3K4me3 and DNase were more highly enriched in full-length promoter-1 and its cropped fragments than in full-length promoter-2 and its cropped fragments. Based on these data, we argued that promoter-1 was more likely the P311 gene promoter, but this finding required further validation through biological experiments.

### Identification of a Novel P311 Promoter With the Dual-Luciferase Reporter Assay

To functionally identify the human P311 gene promoter, promoter-1 and promoter-2 were cloned into the pGL3-Basic reporter vector (Figure 1C). We found that only vector containing promoter-2, and not vector containing promoter-1, had significant promoter activity (Figures 1D,E). Because the average size of a core promoter is 100–200 base pairs, we truncated promoter-1 into 3 fragments for further validation (Figure 1C) and found that none of the fragments showed activity, which was in contrast to the notion of prediction via epigenetic modifications (Figures 1A,B). Two truncated DNA fragments of promoter-2, promoter 2-2 (pro-2-2) and promoter 2-3 (pro-2-3), were cloned into the pGL3-basic vector.

Comparing with pGL3-Basic, the luciferase activities of 293T cells transfected with pro-2-1, pro-2-2, and pro-2-3 have increased by 76, 73, and 623% (Figure 1E). The results suggested that all the truncated fragments had higher luciferase activity, whereas cells transfected with pro-2-3 had the highest activity among all fragment-transfected cells (Figure 1E). To further figure out the core promoter, we truncated pro-2-3 into 0.8-kb (pro-0.8), 0.6-kb (pro-0.6), 0.4-kb (pro-0.4), and 0.2-kb (pro-0.2) fragments, as shown in Figure 1C. The luciferase activities in cells transfected with these truncated fragments, especially pro-0.4, which was identified as the *bona fide* core promoter of the human P311 gene, were significantly higher than those in cells transfected with pro-2-3 (Figure 1F).

### Smad2/3/4 Do Not Directly Bind to TFs of P311 Promoter

**FIGURE 2:**
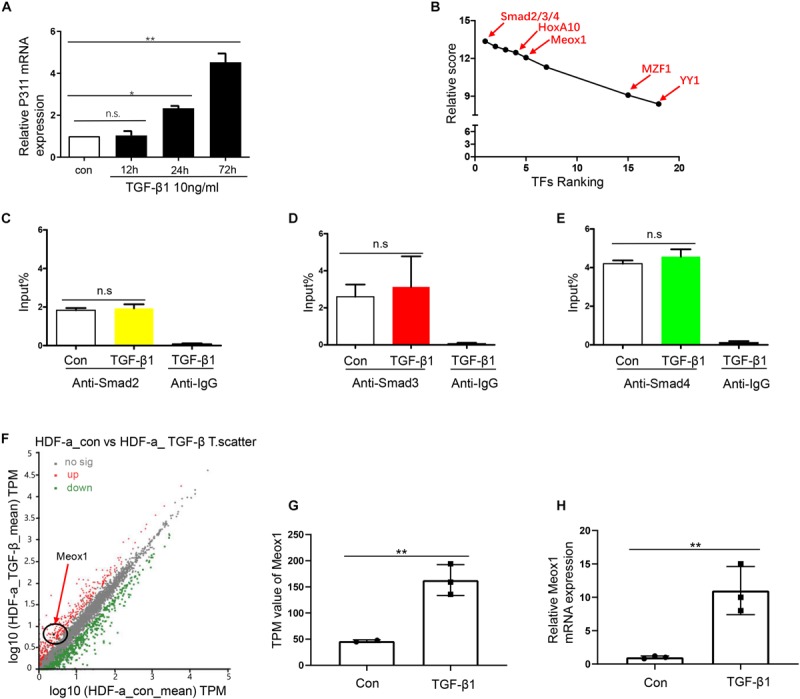
Smad2/3/4 were not the directly bind to TF of P311 promoter. **(A)** Relative mRNA expression of the P311 gene in TGF-β1–stimulated HDF-a cells at different time points. **(B)** The ranks of the predicted TFs by RNA-seq data and relative scores determined with the JASPAR database. **(D,E)** A ChIP-qPCR assay was performed to test the enrichment of Smad2 **(C)**, Smad3 **(D)** or Smad4 **(E)** in the pro-0.4 locus in HDF-a cells under conditions of TGF-β1 stimulation. Anti-IgG was used as a negative control. **(F)** Scatter plots of upregulated and downregulated mRNAs in normal HDF-a cells (*x*-axis) and TGF-β1–stimulated HDF-a cells (*y*-axis). Each point in the figure represents an independent gene. The red points represent significantly upregulated mRNAs in the TGF-β1–stimulated groups. The black points represent mRNAs that were not significantly expressed in stimulated groups and normal groups. The green points represent significantly downregulated mRNAs in the TGF-β1–stimulated groups. The black circle indicates Meox1, which was upregulated. **(G)** The transcripts per kilobase of exon per million mapped reads (TPM) value of the Meox1 gene in TGF-β1–stimulated HDF-a cells compared to normal HDF-a cells determined by RNA-seq. **(H)** Relative mRNA expression of Meox1 in TGF-β1–stimulated and normal HDF-a cells. Con refers to HDF-a cells without TGF-β1 stimulation (con); TGF-β1 refers to HDF-a cells within TGF-β1 stimulation (TGF-β1).

As TFs are important participants in the activation of gene promoters (Wittkopp and Kalay, 2011), we aimed to determine the key TF of pro-0.4. Stimulation of the TGF-β1 pathway can induce many TFs, which play a critical role by binding to the target gene promoters (Kaur et al., 2018). Because we validated that TGF-β1 obviously increased P311 expression after stimulation for 24 h (Figure 2A), we combined the results of predication with JASPAR software^4^ and RNA-seq data from HDF-a cells with or without TGF-β1 stimulation to rank potential TFs of pro-0.4 (Figure 2B). Our data indicated that Smad2/3/4 were candidate TFs that may participate in activation of the P311 promoter for the following two reasons: their higher relative scores and because they are classic downstream TFs of TGF-β1. However, the ChIP-qPCR results showed that nor did Smad2/3/4 specifically alter its enrichment on pro-0.4 after TGF-β1 stimulation. These data revealed that Smad2/3/4 could not directly bind to P311 promoter (Figures 2C–E). Thus, we transmitted our focus on other TFs and focused on Meox1 based on a previous study reporting that TGF-β1–induced Meox1 played vital roles in smooth muscle cell (SMC) differentiation (Wu et al., 2018) and its higher ranking following JASPAR prediction (Figure 2B). The RNA-seq and RT-qPCR results showed increased TGF-β1–induced Meox1 (Figures 2F–H).

### TGF-β1–Induced Meox1 Is Essential for Transcriptional Activity of the P311 Gene

**FIGURE 3:**
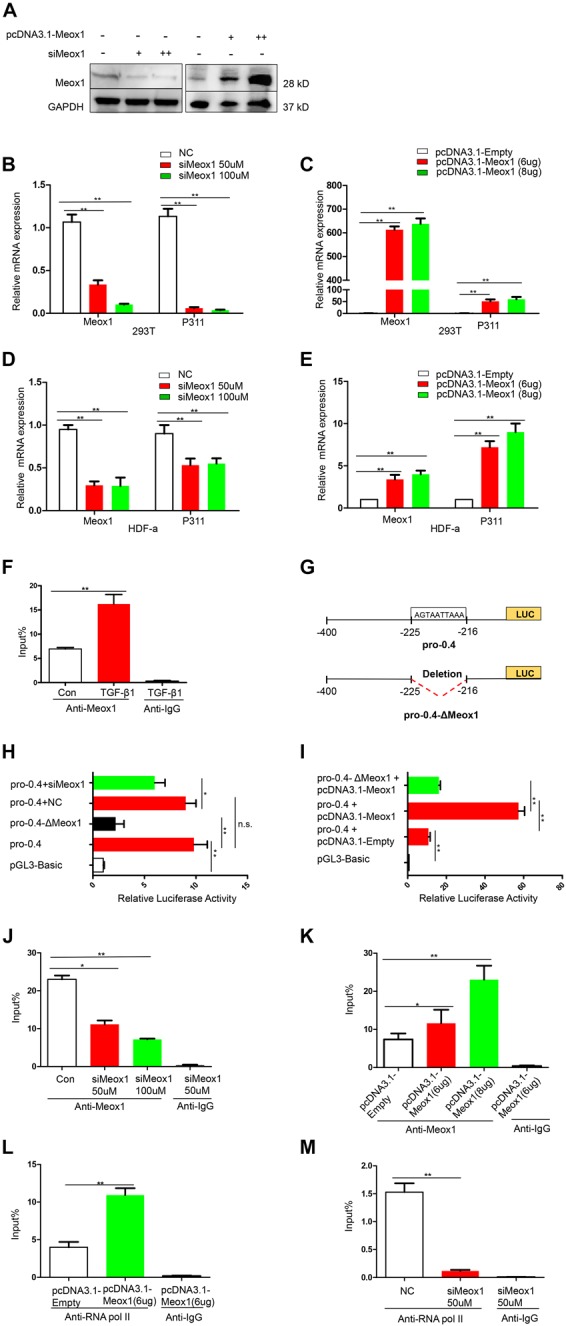
Meox1 was essential for P311 gene transcriptional activity. **(A)** Meox1 protein expression in HDF-a cells following the knockdown (siMeox1) or overexpression (pcDNA3.1-Meox1) of Meox1 determined by Western blotting. GAPDH was used as an internal control. **(B,C)** The relative mRNA expression of P311 and Meox1 in 293T cells in which Meox1 was knocked down or overexpressed determined by RT-qPCR. Scramble siRNA (NC) and the pcDNA3.1 vector (pcDNA3.1-Empty) were used as negative controls. **(D,E)** The relative mRNA expression of P311 and Meox1 in HDF-a cells in which Meox1 was knocked down or overexpressed determined by RT-qPCR. **(F)** ChIP-qPCR assays were used to test the enrichment of Meox1 in the pro-0.4 locus in HDF-a cells with or without TGF-β1 stimulation. Anti-IgG was used as a negative control. **(G)** Detailed information on Meox1-binding site-deficient pro-0.4 (pro-0.4-ΔMeox1). **(H)** The relative luciferase activities in 293T cells cotransfected with siMeox1 and pro-0.4-ΔMeox1 or pro-0.4. NC represent scramble siRNA. **(I)** The relative luciferase activities in 293T cells overexpressing Meox1 transfected with pro-0.4 or pro-0.4-ΔMeox1. The pcDNA3.1 vector (pcDNA3.1-Empty) was used for as an empty control vector. **(J,K)** ChIP-qPCR assays were used to test the enrichment of Meox1 in the pro-0.4 locus of HDF-a cells transfected with siMeox1 or pcDNA3.1-Meox1. **(L,M)** ChIP-qPCR assays were used to test the enrichment of RNA pol II in the pro-0.4 locus in HDF-a cells in which Meox1 was knocked down or overexpressed.

To determine whether Meox1 affects P311 mRNA expression, Meox1 was overexpressed and knocked down in 293T cells and HDF-a cells, which resulted in the increased and decreased expression of P311, respectively (Figures 3A–E), suggesting that Meox1 is a positive regulator of P311. To further uncover the mechanism between them, we compared Meox1 enrichment on promoter-2 with TGF-β1 stimulation. Our results suggested that the enrichment of Meox1 on pro-0.4 locus increased by 1.34-fold after TGF-β1 stimulation (Figure 3F). Then, we deleted the Meox1-binding site in pro-0.4 (pro-0.4-ΔMeox1) to further confirm that Meox1 is a positive TF of P311 (Figure 3G).

Comparing with pro-0.4, the luciferase activity of pro-0.4-ΔMeox1 obviously decreased by 78% (Figure 3H). In addition, the knockdown of Meox1 also declined its luciferase activity of pro-0.4 falling to 34% (Figure 3H). In contrast, the forced expression of Meox1 significantly increased the activity of pro-0.4 by 540% (Figure 3I).

We then examined whether the activity of pro-0.4 was correlated with Meox1 binding to the promoter. The results showed that the trend in the change in P311 mRNA expression was consistent with that in the change in Meox1 enrichment on pro-0.4 (Figures 3J,K). However, we detected only P311 mRNA expression at steady stage using RT-qPCR, which could not quantify P311 transcription. Generally, the enrichment of RNA polymerase II (RNA pol II) on a specific gene promoter represents the real transcriptional level of gene (Kadonaga, 2012). Therefore, we detected the enrichment of RNA pol II on promoter-0.4. Our results indicated that the enrichment of RNA pol II on the P311 gene promoter increased by 1.73-fold with forced expression of Meox1 (Figure 3L), and that decreased by 11.97-fold with knockdown of Meox1 (Figure 3M). In summary, our results suggested that Meox1 contributes to the transcription of P311.

### Meox1 Promoted HDF-a Migration and Proliferation *in vitro*

**FIGURE 4:**
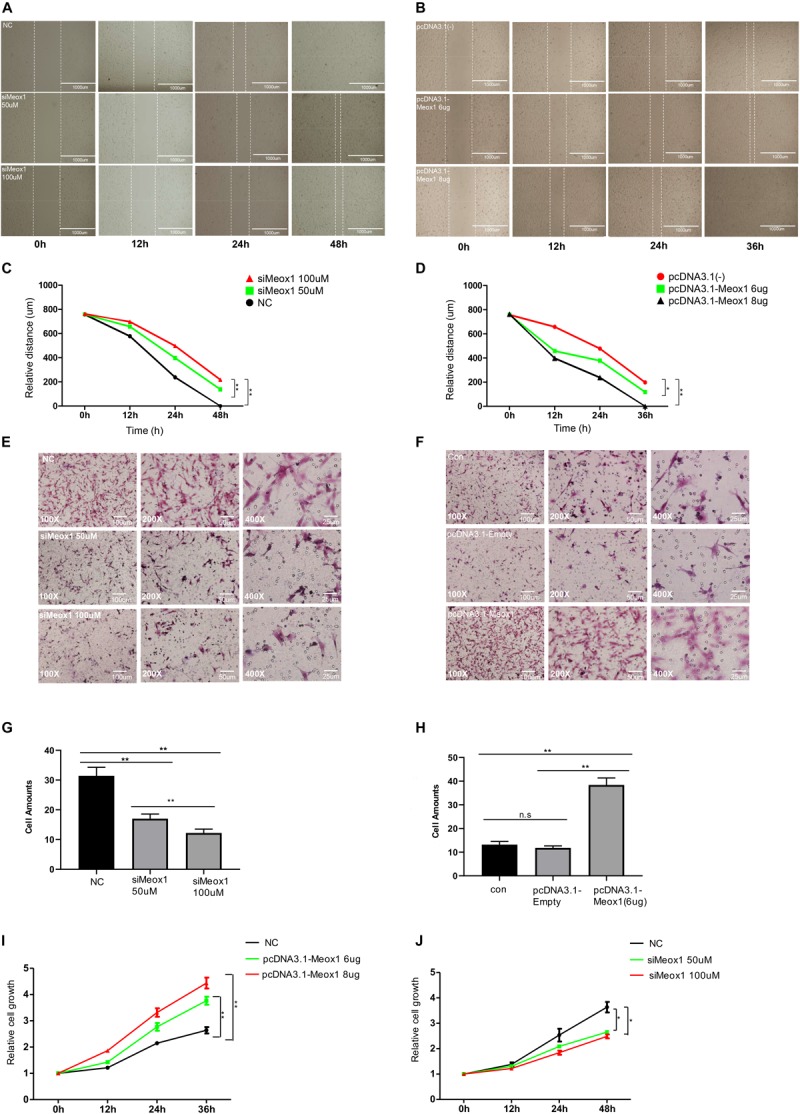
Meox1 promotes HDF-a migration and proliferation *in vitro*. **(A,B)** Cell migration at 12, 24, and 48 h after wound formation was measured using wound-healing assays (scale bar, 400 μm). Cells transfected with mimics NC, 50 μM Meox1 siRNAs, and 100 μM siMeox1 **(A)** or pcDNA3.1-Empty and 6 or 8 μg of pcDNA3.1-Meox1 **(B)** were assessed by wound-healing assay. **(C,D)** Analysis of wound-healing distance in the siMeox1 and pcDNA3.1-Meox1 groups determined by wound-healing assays. **(E,F)** Migration of HDF-a cells transfected with mimics NC, 50 μM Meox1 siRNAs, and 100 μM siMeox1 **(E)** or pcDNA3.1-Empty and 6 or 8 μg of pcDNA3.1-Meox1 **(F)** were assessed by Transwell migration assay (scale bars, 100, 50, and 25 μm). **(G,H)** Analysis of the number of migrated cells in the siMeox1 **(G)** and pcDNA3.1-Meox1 **(H)** groups determined by Transwell migration assays. **(I,J)** CCK8 assays were performed to analyze cell proliferation.

Meox1 was identified as a key TF of P311 gene expression. P311 gene expression was found to be dramatically increased in hypertrophic scars (Wu et al., 2004), and fibroblast cells are the main constituents of hypertrophic scars, so it was necessary to determine the roles of Meox1 in fibroblast cells. To address the effects of Meox1 in cell migration, wound-healing assays and Transwell assays were performed. For wound-healing assays, Meox1 knockdown reduced the scratch healing width, whereas its forced expression increased the width of scratch healing in fibroblasts (Figures 4A–D). For Transwell assays, the knockdown of Meox1 significantly decreased the numbers of migrated fibroblasts (Figures 4E,G). In addition, forced expression of Meox1 dramatically increased the numbers of migrated fibroblasts (Figures 4F,H). *In vitro* experiments results suggested that Meox1 significantly increased the cell migration of fibroblast cells (Figures 4A–H). In addition, CCK8 assays demonstrated that the forced expression of Meox1 significantly promoted the proliferation of human fibroblast cells and that Meox1 knockdown inhibited the proliferation of human fibroblast cells (Figures 4I,J).

## Discussion

In this study, we first identified a novel promoter for the human P311 gene at chr5:111999065–111999465. Furthermore, Meox1 was significantly induced by TGF-β1 and then bound this P311 promoter, acting as an active transregulator that increased the binding of RNA Pol II to the P311 promoter. Subsequently, Meox1 itself dramatically promoted HDF-a migration and proliferation.

In locating the novel P311 promoter, epigenetic modifications, such as DNA methylation and histone modifications, are the initial and key factor for the regulation of gene expression (Heintzman et al., 2007). Histone modification on chromatin alters gene expression through remolding chromatin organization and by determining the binding of transregulators to DNA, whereas DNA methylation variation often related to gene “on or off” (Kouzarides, 2007). Heintzman et al. (2007) reported that DNase I, H3K4me1, H3K4me3, and K3K27ac were often enriched at active promoters. Roadmap epigenetic projects collected all genome-wide maps of histone modifications, DNA accessibility, DNA methylation, and RNA expression of human cells (Mullard, 2015). Thus, we took advantage of this online database to review epigenetic markers for the potential promoters of P311 gene, suggesting that promoter-1 was more likely to be the promoter. However, we finally defined the promoter-2, rather than promoter-1, was a *bona fide* promoter for P311. Therefore, the epigenetic data only give us the initial evidence. What matters most are biological verification experiments. Nevertheless, we only determined the luciferase activities through inserting the potential promoter into pGL3 vectors. Thus, we need to perform *in vivo* knockdown experiments to confirm results with CRISPR/Cas9.

*Meox1* is a subfamily of HOX genes, including Meoxl and Meox2 (Candia et al., 1992). According to the ranking of TFs, Meox1 was ranked at a higher position and was regarded as a target of TGF-β1. Wu et al. reported that Meox1 was essential and was up-regulated for TGF-β1 induced for SMC differentiation (Wu et al., 2018). Rodrigo et al. (2004) reported that Meox1 was important for Bapx1 expression and transactivated by direct binding to the bagpipe homeobox homolog 1 (Bapx1) promoter in the sclerotome. Considering these factors, we argued that Meox1 was more likely to be a positive transregulator in P311 regulation. And our results suggested that Meox1 was important for transcriptional expression for P311 via directly binding P311 promoter. Previous studies have shown that Meox1 is critical for pathological process of cardiomyopathy and heart failure through accelerating myocardial hypertrophic decompensation by GATA binding protein 4 (Gata4) (Lu et al., 2018). Sun et al. (2019) suggested that Meox1 regulated breast cancer stem cells and mesenchymal-like cell proliferation. In addition, Meox1 was found to promote SMC phenotypic modulation and differentiation (Wu et al., 2018). For function assays, our results demonstrated that Meox1 was a key factor for migration and proliferation in fibroblasts. Meox1 is likely to act on the upstream of P311; our results only indicated the independent function of Meox1 in fibroblasts. We still need to construct Meox1-binding site knockout (KO) or promoter KO human fibroblast cell line to confirm the relationship between Meox1 and P311 *in vivo*.

Besides Meox1, other TFs might mediate the P311 promoter such as HoxA10, which is a highly conserved homeodomain TF that plays a pivotal role in embryogenesis and definitive hematopoiesis (Acampora et al., 1989). It was reported to activate fibroblast growth factor 2 (FGF2) through binding to the *cis*-elements of FGF2 locus. In addition, HoxA10 was highly ranked in our study, so we propose that HoxA10 is an important candidate TF of P311 (Figure 2B). However, the detailed mechanisms of P311 gene transcriptional regulation remain unclear.

In summary, in this article, we first identified a novel promoter of P311 and then defined TGF-β1–induced Meox1 as a positive TF that significantly increased the migration and proliferation of human fibroblast cells via binding to the P311 promoter. Our findings provide guidelines for exploring P311 gene transcriptional regulation and fill a gap in knowledge regarding P311 transcriptional regulation. Furthermore, Meox1 may be a target for clinical fibrosis treatment in the future.

## Data Availability Statement

Publicly available datasets were analyzed in this study. This data can be found here: PRJNA590308.

## Author Contributions

ZW, HL, CH, and JZ: investigation. ZW and YT: data curation. ZW: writing—original draft preparation. YT and GL: writing—review and editing. HD: methodology supervision. WH, YW, YT, and GL: study supervision. GL: funding acquisition.

## Conflict of Interest

The authors declare that the research was conducted in the absence of any commercial or financial relationships that could be construed as a potential conflict of interest.

## Footnotes

1 jaspar.genereg.net

2 genome.ucsc.edu

3 http://www.roadmapepigenomics.org/

4 www.jaspar.com
