# Supplementary material for: Molecular Mechanism of Mesenchyme Homeobox 1 in Transforming Growth Factor β1–Induced P311 Gene Transcription in Fibrosis
Source: Front Mol Biosci. 2020 Apr 28;7:59. doi: 10.3389/fmolb.2020.00059 (PMC7199492; doi:10.3389/fmolb.2020.00059)
Supplement: Supplementary file 1 [file Data_Sheet_1.docx]

**Supplementary Figure and Tables**

**
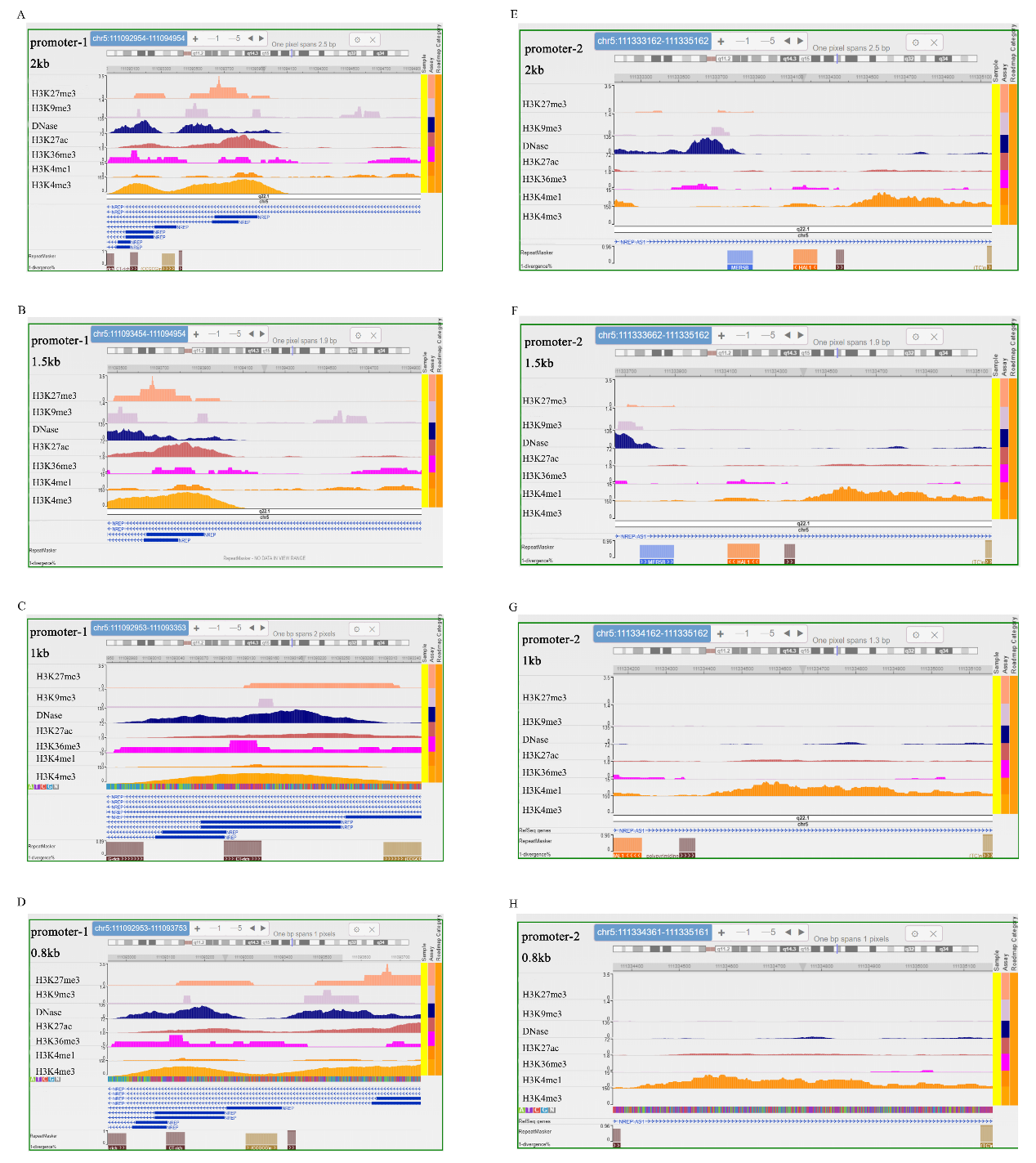
**

**Figure S1 A-H.** Levels of epigenetic modifications of two full length promoter and its cropped fragments of P311: promoter-1 2kb (A), promoter-1 1.5kb (B), promoter-1 1kb (C), promoter-1 0.8kb (D), promoter-2 2kb (E), promoter-2 1.5kb (F), promoter-2 1kb (G) and promoter-2 0.8kb (H) determined with the Roadmap Epigenomics Project database.

**Table S1. Primers of promoter1 related vectors**

| **Vector name** | **Forward / Reverse primers** |
| --- | --- |
| pro-1-1 | F: GGGGTACCCC AGCATGAACTCTGCAGGGTTG  R: CCGCTCGAGCGG GAAATTCAGAATAGTGTTGGC |
| pro-1-2 | F: GGGGTACCCC CCAGCCTTGCTGCCACTGTG  R: CCGCTCGAGCGG TTTTGCAGGAAAATTACTGTGATG |
| pro-1-3 | F: GGGGTACCCC GAGCGAAGCAGAAAATGGTG  R: CCGCTCGAGCGG TGCAGGAAAATTACTGTGATG |
| pro-1-4 | F: GGGGTACCCC TGTAAACTCGTCGTTCTTAT  R: CCGCTCGAGCGG TTGCAGGAAAATTACTGTGA |

**Table S2. Primers of promoter2 related vectors**

| **Vector name** | **Forward / Reverse primers** | |
| --- | --- | --- |
| pro-2-1 | F: GGGGTACCCC GAGCATAACTAACCCTGCTGGTCAG  R: TCCCCCGGGGGA GAGAGAGAGAGAGAGAAAGAGAGAG | |
| pro-2-2 | F: GGGGTACCCC GAGCATAACTAACCCTGCTGGTCAG  R: TCCCCCGGGGGA CAGAATAGTGTTGGCAAGAGAG | |
| pro-2-3 | F: GGGGTACCCC CTGTTACCTCCTTTTCATCT  R: TCCCCCGGGGGA GAAATTCAGAATAGTGTTGGC | |
| pro-0.8 | F: GGGGTACCCC TCCTTTTAATAGATAATCACCAC  R: TCCCCCGGGGGA GAAATTCAGAATAGTGTTGGC |  |
| pro-0.6 | F: GGGGTACCCC AGGCTACTTTTAGGTTCTATGT  R: TCCCCCGGGGGA AAATTCAGAATAGTGTTGGC | |
| pro-0.4 | F: GGGGTACCCC AGCAGAGTGCTCTTTTT  R: TCCCCCGGGGGA AAATTCAGAATAGTGTTGGC | |
| pro-0.2 | F: GGGGTACCCC CTGTTTAATTACTGTGATGGCT  R: TCCCCCGGGGGA TCAGAATAGTGTTGGCAAGA | |

**Table S3. siRNA target sequences**

| **Target interfered gene** | **Target sequence** |
| --- | --- |
| Smad2 | 5’ GGAGTGCGCTTATACTACA+dTdT 3’ |
| Smad3 | 5’ GGTGCTCCATCTCCTACTA+dTdT 3’ |
| Smad4 | 5’ GGATTGAAATTCACTTACA+dTdT 3’ |
| Meox1 | 5’ CTGCCAATGAGACAGAGAA+dTdT 3’ |

**Table S4. RT-qPCR Primers of target gene**

| **Gene name** | **Forward / Reverse primers** |
| --- | --- |
| Meox1 | F: GGCAGCGTACCCTGACTTC  R: GGTCCCCATTTCCTTGGAACC |
| P311 | F: TCCAAACAAGGACATGGAGGG  R: AGGTAACTGATTCTTGGGGAG |
| GAPDH | F: GGTCGGAGTCAACGGATTTG  R: TGGGTGGAATCATATTGGAAC |
| Smad2 | F: CGTCCATCTTGCCATTCACG  R: CTCAAGCTCATCTAATCGTCCTG |
| Smad3 | F: CCATCTCCTACTACGAGCTGAA  R: CACTGCTGCATTCCTGTTGAC |
| Smad4 | F: CTCATGTGATCTATGCCCGTC  R: AGGTGATACAACTCGTTCGTAGT |

**Table S5. ChIP-qPCR Primers for pro-0.4 locus**

| **primer name** | **Forward / Reverse primers** |
| --- | --- |
| 0.4-a (position-1) | F: TTGCTCAGACCCGAAAGAGA  R: CCCAAGCTGAGAGTGACCTA |
| 0.4-2-b (position-2) | F: GGGCATCTCAAGTCAGTCCA  R: AGGAGATCAAGTGACAGCCA |
| 0.4-3-c (position-3) | F: TAGGTCACTCTCAGCTTGGG  R: AGAATGTCATCAGCCGGTAG |
